# Genipin induces mitochondrial dysfunction and apoptosis via downregulation of Stat3/mcl-1 pathway in gastric cancer

**DOI:** 10.1186/s12885-019-5957-x

**Published:** 2019-07-27

**Authors:** Min Jee Jo, Soyeon Jeong, Hye Kyeong Yun, Dae Yeong Kim, Bo Ram Kim, Jung Lim Kim, Yoo Jin Na, Seong Hye Park, Yoon A. Jeong, Bu Gyeom Kim, Hassan Ashktorab, Duane T. Smoot, Jun Young Heo, Jeongsu Han, Dae-Hee Lee, Sang Cheul Oh

**Affiliations:** 1 Division of Oncology/Hematology, Department of Internal Medicine, Korea University Guro Hospital, 148, Gurodong-gil, Guro-gu, Seoul, 08308 Republic of Korea; 2 Graduate School of Medicine, College of Medicine, Korea University, Seoul, 08308 Republic of Korea; 3 Department of Medicine, Howard University, Washington, DC 20060 USA; 4 Department of Medicine, Meharry Medical Center, Nashville, TN 37208 USA; 5 Department of Medical Science, School of Medicine, Chung-nam National University, Munhwa-dong, Jung-gu, Daejeon, 301-747 Republic of Korea

**Keywords:** Genipin, Mcl-1, Stat3, Mitochondrial dysfunction, Apoptosis, Gastric cancer

## Abstract

**Background:**

Genipin is a compound derived from gardenia fruit extract. Although Genipin has anti-tumor effects in various cancers, its effect and mechanism in gastric cancer remain unclear. Here, we investigated the relationship between the anticancer effect of Genipin and signal transducer and activator of transcription (Stat3)/myeloid cell leukemia-1 (Mcl-1) in human gastric cancers.

**Methods:**

MTT assays were performed to determine the cell viability of gastric cancer and gastric epithelial cell lines (AGS, MKN45, SNU638, MKN74, HFE-145). A TUNEL assay and Western blotting were carried out to investigate apoptosis. Stat3 activity was measured by proteome profiler phospho kinase array, immunofluorescence and immunoblotting. Mitochondria function was monitored with an XF24 analyzer and by flow cytometry, confocal microscopy using fluorescent probes for general mitochondrial membrane potential (MMP).

**Results:**

Genipin induced apoptosis in gastric cancer cells, including AGS and MKN45 cells. Genipin also reduced Mcl-1 mRNA and protein levels. Furthermore, we found that phosphorylation of Stat3 is regulated by Genipin. Additionally, the protein level of phospho Janus kinase 2 (JAK2) was decreased by Genipin treatment, indicating that the Stat3/JAK2/Mcl-1 pathway is suppressed by Genipin treatment in gastric cancer cells. Mcl-1 is closely related to mitochondrial function. These findings suggest that Genipin contributes to the collapse of mitochondrial functions like MMP.

**Conclusions:**

Genipin induced apoptosis by suppressing the Stat3/Mcl-1 pathway and led to mitochondrial dysfunction. Our results reveal a novel mechanism for the anti-cancer effect of Genipin in gastric cancer.

**Electronic supplementary material:**

The online version of this article (10.1186/s12885-019-5957-x) contains supplementary material, which is available to authorized users.

## Background

Cancer is a major cause of human death, and chemotherapy drugs have been developed to improve the survival rate of patients with cancer, but these drugs show various side effects. The main strategy in cancer therapy involving chemotherapy drugs is to induce apoptotic cell death [1]. Apoptosis is a programmed cellular process that induces cell death [2]. Myeloid cell leukemia-1 (Mcl-1), an anti-apoptotic B-cell lymphoma 2 (Bcl-2) family member, is essential for apoptosis [3]. Mcl-1 blocks apoptosis by binding to and dissociating from Bak and Bax, which are pro-apoptotic Bcl-2 family members that form mitochondrial permeability transition pores in the mitochondrial membrane to induce cytochrome c release into the cytoplasm, electron transport change, and decreased mitochondrial membrane potential (MMP) [4, 5].

The Janus kinase (JAK)/signal transducer and activator of transcription (Stat) signaling regulates various processes such as cell growth, survival, angiogenesis, and immunity and are activated by growth factors and cytokines [6, 7]. When ligands bind to the receptor, a conformational change occurs to activate JAKs. Activated JAKs phosphorylate their receptors and Stats, and the phosphorylated Stats are then released from the receptor, dimerized, and translocated to the nucleus to induce the transcription of the target gene [8]. Aberrant activation of Stat3 plays an important role in the growth and development of human cancers, including breast [9], lung [10], and colorectal cancers [11]. Stat3 is a major regulator of Mcl-1 expression. For example, the activation of autocrine interleukin 6 (IL-6) and protein kinase C activation by nuclear factor-κB upregulated Stat3, and then increased Mcl-1 gene expression [12].

Genipin is a natural constituent of *Gardenia jasminoides*, which regulates various cellular processes, including proliferation [13], death [14], angiogenesis [15], oxidative stress [16], and inflammation [17]. Genipin has shown anticancer effects in various cancers such as gastric, cervical, breast, and lung cancers [18–21]. However, its mechanism of action in gastric cancer cells remains unclear.

We previously reported that Genipin attenuates sonic hedgehog signaling through the p53-dependent regulation of Noxa, a pro-apoptotic Bcl-2 family protein in colorectal cancer. In this study, we focused on the role of Mcl-1 in apoptosis by Genipin. We demonstrate here for the first time that Genipin causes apoptotic cell death by Mcl-1. Our data showed that Genipin decreased cell viability and increased apoptosis. In addition, Genipin-induced cell death was associated with JAK2/Stat3 and Mcl-1 inhibition. Taken together, these results implicate Genipin in the induction of apoptotic cell death via JAK2/Stat3-regulated Mcl-1 suppression, suggesting that Genipin can potentially be an effective therapy for treating gastric cancer.

## Methods

### Cell culture

The human gastric carcinoma AGS, MKN74, and MKN45 cell lines were purchased from the American Type Culture Collection (Manassas, VA, USA) and maintained according to the manufacturer’s instructions. The human gastric epithelial HFE-145 cell line were obtained from Hassan’ laboratory. The human gastric carcinoma SNU638 cell line was obtained from the Korean Cell Line Bank (Seoul, Korea). We have confirmed the cell lines used in the experiments with specialized STR profiling and tested for mycoplasma contamination. The gastric carcinoma cell lines were grown in RPMI1640 medium (Gibco, Grand Island, NY, USA) containing 10% fetal bovine serum (FBS, Sigma, St. Louis, MO, USA) with 100 mg/mL penicillin and streptomycin (P/S, GenDEPOT, Barker, TX, USA) and normal cell lines were grown in Dulbecco’s Modified Eagle’s Medium (DMEM, GenDEPOT, Barker, TX, USA) containing 10% FBS with 100 mg/mL P/S.

### Transfection

Cells were seeded and incubated at 37 °C overnight. For RNA interference, the cells were incubated with small interfering RNA (siRNA) and Lipofectamine RNAiMAX 2000 (Invitrogen, Carlsbad, CA, USA) in OPTI-MEM reduced serum medium (Life Technologies, Carlsbad, CA, USA) for 6 h. Following incubation, the cells were washed, and the medium was replaced with fresh culture medium. Mcl-1 siRNA (siMcl-1) and Stat3 siRNA (siStat3) were obtained from Santa Cruz Biotechnology (Dallas, TX, USA).

To overexpress the target gene, His-Mcl-1 plasmid was incubated on cells with containing the His-Mcl-1 plasmid were incubated with Lipofectamine 2000 (Invitrogen). After 6 h of incubation at 37 °C, the medium was replaced with fresh culture medium.

### Reagents and antibodies

Genipin was purchased from Cayman Chemical (Ann Arbor, MI, USA). Cleaved PARP, caspase 3, cleaved caspase 8, caspase 9, Bax, Bim, Noxa, p53 upregulated modulator of apoptosis, Bid, Mcl-1, X-linked inhibitor of apoptosis, Stat3, phospho-Stat3, JAK2, phospho-JAK2, and voltage-dependent anion channel (VDAC), Snai1 antibodies were all purchased from Cell Signaling Technology (Danvers, MA, USA). Bcl-2, B-cell lymphoma extra-large (Bcl-xL), survivin, NADH dehydrogenase (ubiquinone) 1 alpha subcomplex subunit 9 (NDUFA9), succinate dehydrogenase complex flavoprotein subunit A (SDHA), Rieske iron-sulfur (RieskeFeS), cytochrome c oxidase I, and ATP synthase subunit alpha (ATP5A) antibodies were purchased from Santa Cruz Biotechnology. Vimentin was purchased from DAKO (Brüsseler Str. Berlin, German). E-cadherin and N-cadherin were purchased from BD Biosciences (Franklin Lakes, New Jersey, USA). For secondary antibodies, anti-mouse-IgG-horseradish peroxidase (HRP) and anti-rabbit-IgG-HRP were purchased from Cell Signaling Technology. Z-VAD-FMK, a caspase inhibitor, was purchased from Promega (Madison, WI, USA). Ruxolitinib, a JAK2 inhibitor, was purchased from Sigma.

### Cell proliferation assay

Cell proliferation was determined by thiazolyl blue tetrazolium bromide (MTT, Sigma) assay. Viable cells convert MTT to insoluble formazan crystals. Cells were seeded at a density of 1 × 10^4^ cells per well in 96-well plates. The cells were treated with Genipin for 24 h and subsequently with MTT solution for 4 h at 37 °C. The absorbance at 595 nm was measured using a microplate reader (SPECTRA190, Molecular Devices, Sunnydale, CA, USA).

### Colony formation assay

Cells were seeded into 6-well plates at a low density of approximately 1 × 10^3^ cells per well. Cells were cultured for 14 days. The plates were washed with phosphate-buffered saline (PBS) and stained with crystal violet. Colony formation images were captured with a camera. The number of colonies was scored using Image J software (NIH, Bethesda, MD, USA).

### Apoptosis analysis (flow cytometry)

One of the earliest features of apoptosis is the translocation of phosphatidylserine from the inner to outer leaflet of the plasma membrane, which can be detected by the binding of Annexin V [22]. Apoptosis was analyzed with an Annexin V-Fluorescein isothiocyanate Apoptosis Detection kit (BioBud, Seoul, Korea). The cells were untreated or treated with Genipin for 24 h and then trypsinized and centrifuged at 2000 rpm for 5 min. The cells were resuspended in binding buffer, and then stained with 1.25 μL Annexin V-fluorescein isothiocyanate reagent and 10 μL propidium iodide (PI) reagent for 30 min at room temperature (RT) in the dark. Moreover, to measure the cell cycle, harvested cells were stained with PI for 30 min at RT. Staining was then terminated, and the cells were immediately analyzed by flow cytometry (Beckman Coulter, Brea, CA, USA).

### TdT-mediated dUTP nick-end labeling (TUNEL) assay

The cells on the coverslip treated with Genipin were fixed with 4% paraformaldehyde and permeabilized with 0.5% Triton-X 100. Next, the cells were stained using the In Situ Cell Death Detection kit (Roche, Basel, Switzerland). DNA fragmentation was visualized by TUNEL assay according to the manufacturer’s instructions. Finally, fluorescence images were obtained using a confocal microscope (Carl Zeiss, Oberkochen, Germany).

### Reverse transcription-polymerase chain reaction (RT-PCR)

Total RNA extraction was performed using TRIZOL reagent (Life Technologies) according to the manufacturer’s instructions. Transcript amplification was performed using an RT-PCR kit (Life Technologies). PCR amplification was performed using the following primers: Mcl-1, forward: 5′-*GCG ACT GGC AAA GCT TGG CCT CAA*-3′, reverse: 5′-*GTT ACA GCT TGG ATC CCA ACT GCA*-3′, actin, forward: 5′-*ACC CAG ATC ATG TTT GAG AC*-3′, and reverse: 5′-*GGA GTT GAA GGT AGT TTC GT*-3′.

### Quantitative real-time PCR (qRT-PCR)

Total RNA was extracted using TRIZOL reagent (Life Technologies). Transcripts were amplified using an RT-PCR kit (Life Technologies). qRT-PCR was performed on an Applied Biosystems Quantstudio 6Flex qRT-PCR using Taqman probes (Applied Biosystems, Foster City, CA, USA). mRNA expression was normalized to the levels of GAPDH and β-actin.

### Immunoblotting

Western blotting was carried out as previously described [23]. Immunoreactive proteins were visualized using a chemiluminescence protocol (DoGEN ECL, Daeil Lab Service Co. Ltd., Seoul, South Korea).

### Phospho kinase Array kit

Various kinase phosphorylation sites were analyzed using the Proteome Profiler Human Phospho Kinase Array kit (R&D Systems, Minneapolis, MN, USA) according to the manufacturer’s instructions. Briefly, AGS cells were either untreated or treated with Genipin (150 μM). Harvested cells were then incubated with lysis buffer 6 for 30 min on ice and the cell lysate was centrifuged at 15,000 rpm for 5 min. Proteins were quantified by the bicinchoninic acid protein assay. The proteins were incubated with each membrane overnight at 4 °C. Each membrane was washed and incubated with diluted detection antibody cocktail A and B for 2 h at RT. Next, each membrane was washed and incubated with streptavidin-HRP for 30 min at RT. The phosphorylation signal was developed with electrochemiluminescence solution (DoGEN) and recorded on X-ray film.

### Invasion assay

Invasion assay was performed using transwell chamber with 8 μm pores (Corning Incorporated, ME, USA). The upper chamber of the transwell was coated with Matrigel and incubated at 37 °C for 1 h. Then, 5 × 10^6^ cells resuspended with serum free medium was seeded in the upper chamber, and the lower chamber was added with fresh culture medium containing 2% FBS. After incubated for 48 h, the Matrigel on the surface of the upper chamber was wiped off, fixed and stained with crystal violet. The invaded cells were captured with a light microscope and counted.

### MMP assay

MMP was assessed by staining with JC-1(Life Technologies) and tetramethylrhodamine ethyl ester (TMRE, Invitrogen) dyes. The cells were seeded and then each dye was directly added to the cell culture medium. The cells were then incubated for 10 min at 37 °C with the dyes. Finally, the cells were harvested and evaluated by flow cytometry.

### Number of mitochondria

The cells were seeded and then incubated with Mitotracker (Thermo Fisher Scientific, Waltham, MA, USA) and 10-*N*-nonyl acridine orange (NAO, Invitrogen) for 10 min at 37 °C. Following incubation, the cells were fixed and permeabilized with 3.7% formaldehyde and 0.5% Triton X-100 for 15 min at RT, respectively. The cells were washed three times with PBS and then stained with 4′,6-diamidino-2-phenylindole (DAPI) for 10 min at 37 °C. The cells were mounted on coverslips and images were captured using a confocal microscope.

### Oxygen consumption rate (OCR) and extracellular acidification rate (*ECAR*)

The cells were seeded (3 × 10^4^ cells/well) into an XF24 cell culture microplate (Seahorse Bioscience, North Billerica, MA, USA). On the following day, the cells were treated with Genipin (150 μM) prior to any measurements. One hour before the measurements, the culture medium was replaced with XF24 medium containing glucose. The OCR and ECAR was measured using an XF24 extracellular flux analyzer. To validate the measured OCR, oligomycin (2 μg/mL), carbonyl cyanide m-chlorophenyl hydrazome (CCCP) (5 μM), and rotenone (2 μM) were added sequentially.

### Mitochondrial reactive oxygen species (ROS)

AGS cells were seeded in a 6-well plate and Genipin were treated for 24 h. After incubation for 30 min at 37 °C with MitoSOX (Thermo Fisher Scientific), the cells were harvested with trypsin and analyzed for mitochondrial ROS using flow cytometry.

### Immunofluorescence

The cells were incubated at 37 °C overnight, fixed in 3.7% formaldehyde for 15 min at RT, and then washed three times with PBS. Next, the cells were incubated with 0.5% Triton X-100 for 15 min at room temperature. The cells were incubated in blocking buffer (3% bovine serum albumin with PBS) for 1 h at 4 °C, followed by incubation with primary antibody at 4 °C overnight. The cells were washed three times for 5 min, after which Alexa Fluor 488-conjugated goat anti-mouse secondary antibody (Invitrogen, diluted 1:200 in PBS) and Alexa Fluor 594-conjugated goat anti-rabbit secondary antibody (Invitrogen, diluted 1:200 in PBS) were added for 17 min at 4 °C. After three washes with Tris-buffered saline with Tween 20, the cells were mounted and analyzed by confocal microscopy (Carl Zeiss).

### Statistical analysis

Each assay was performed in triplicate and independently repeated at least three times. Statistical analyses were carried out using GraphPad InStat 6 Software (GraphPad, Inc., La Jolla, CA, USA). Statistical significance was defined as *P* values < 0.05 (*, **, and *** means *P* < 0.05, *P* < 0.01, and *P* < 0.001, respectively).

## Results

### Apoptotic effects of Genipin on gastric cancer

**Fig. 1:**
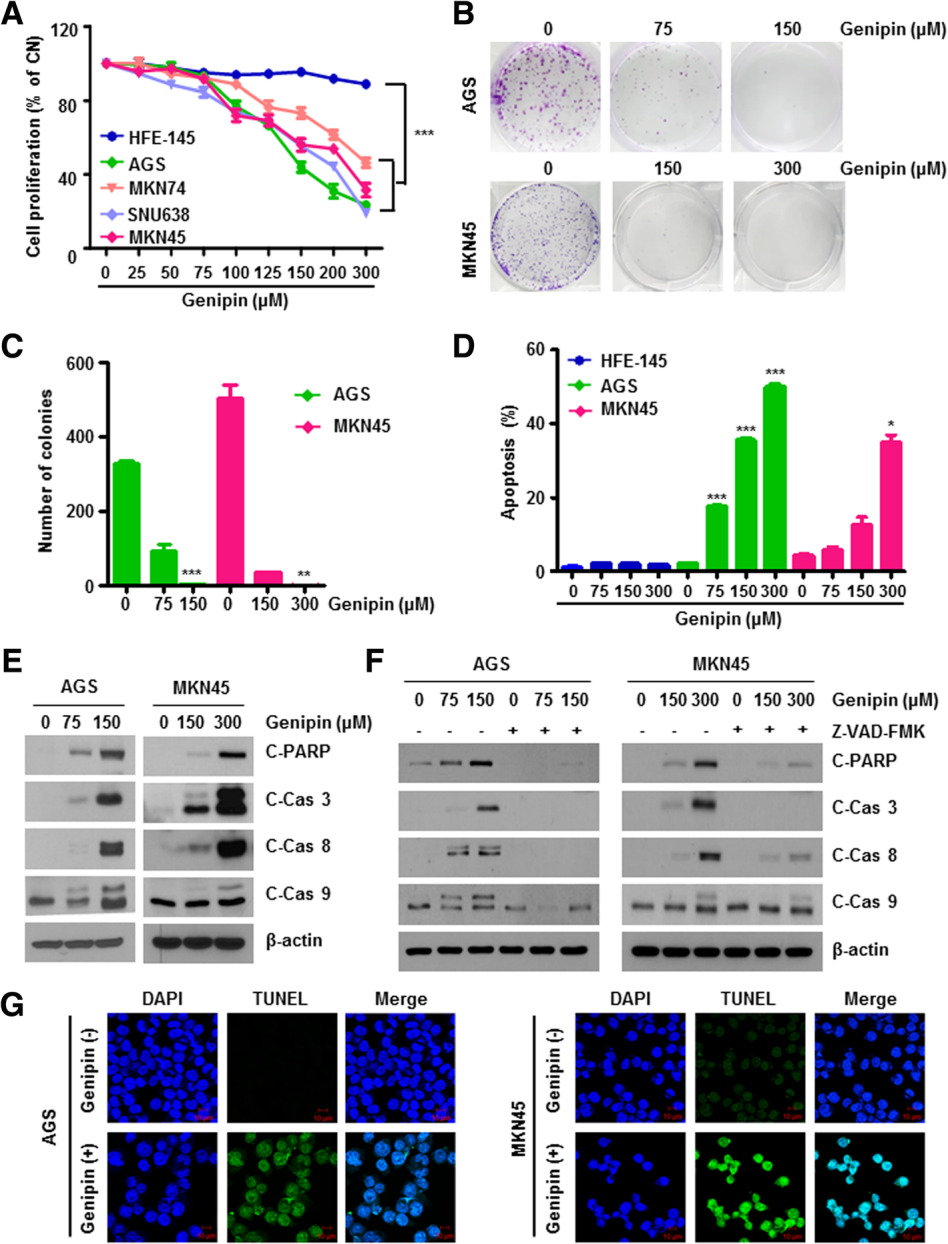
Genipin induces apoptosis in gastric cancer. **a** Cells were treated with different doses of Genipin for 24 h in various gastric cancers and gastric epithelial cell lines. Cell proliferation was evaluated by MTT assay. **b** HFE-145, AGS, and MKN45 cells were treated with the indicated doses of Genipin (0–300 μM) for 24 h. Cell apoptosis was determined by Annexin V/PI staining using flow cytometry. **c**, **d** Colony formation assay of Genipin treatment in AGS (upper) and MKN45 (lower) cells (**c**). The graph represents quantification of colony formation. **, and *** means *P* < 0.01, and *P* < 0.001, respectively (**d**). **e** Cells were treated with 150 μM Genipin for 24 h. Cell lysate was evaluated by western blotting using cleaved PARP, caspase 3, caspase 8, and caspase 9. β-Actin was used as a loading control. **f** Cells were pre-treated with 25 μM Z-VAD-FMK for 30 min. After treatment, the cells were treated with 150 and 300 μM Genipin for 24 h. The protein levels of cleaved PARP, caspase 3, caspase 8, and caspase 9 were detected by western blotting. β-Actin was used as a loading control. **g** Cells were treated with 150 μM Genipin for 24 h. Detection of apoptosis by TUNEL assay in AGS (left) and MKN45 (right) cells using in situ cell death detection kit. Images were captured using a confocal microscope (Scale bar, 10 μm)

To identify the anti-cancer effects of Genipin on gastric cancer cells, we performed cell proliferation analysis after Genipin treatment of various gastric cancer cells such as AGS, MKN45, SNU638, and MKN74. Genipin decreased cell proliferation in a dose-dependent manner in gastric cancer cells compared to in the gastric epithelial HFE-145 cell line (Fig. 1a). To evaluate the clonogenic survival ability of Genipin-treated cells, we conducted a colony formation assay. As shown in Fig. 1b and c, colony formation was attenuated by Genipin exposure.

To investigate whether the decreased viability by Genipin caused apoptosis, we examined the number of Annexin V/PI double-stained cells by flow cytometry. Genipin led to a dose-dependent increase in Annexin V/PI double-positive cells (Fig. 1d). Moreover, Genipin remarkably increased the protein levels of cleaved PARP, caspase 3, caspase 8, and caspase 9 (Fig. 1e), which are well-known apoptotic markers, and TUNEL-positive cells (Fig. 1g). Moreover, Sub-G1 populations were increased in a dose-dependent manner with Genipin (Additional file 1: Figure S1). To determine whether apoptosis by Genipin treatment is caused by the caspase cascade, AGS cells were pre-treated with Z-VAD-FMK, a pan-caspase inhibitor, for 30 min, and then treated with Genipin. Z-VAD-FMK inhibited the increases in cleaved PARP, cleaved caspase 3, cleaved caspase 8, and cleaved caspase 9 protein levels (Fig. 1f), indicating that Genipin enhances apoptosis in gastric cancer cells.

### Genipin mediates apoptosis by downregulating mcl-1

**Fig. 2:**
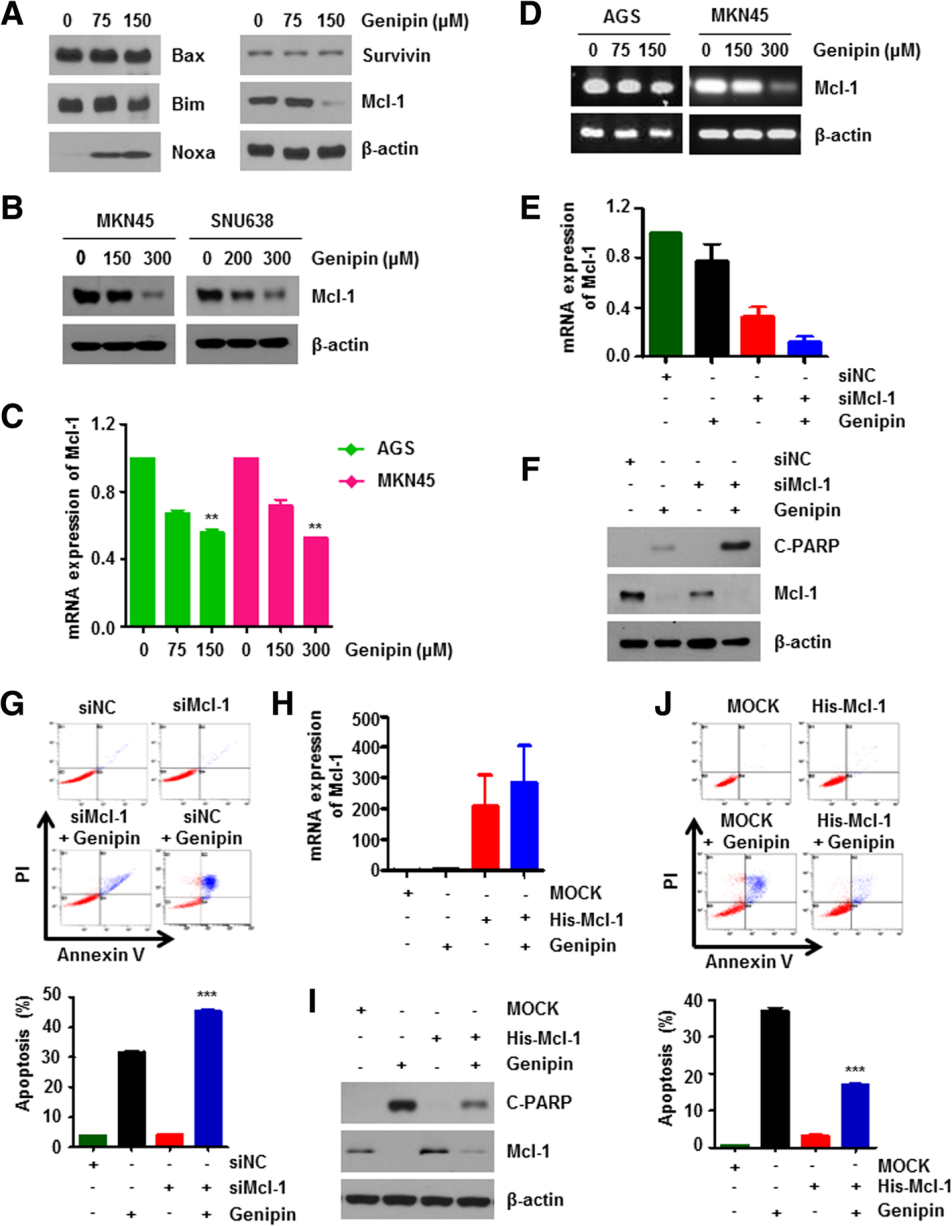
Genipin leads to apoptosis through Mcl-1 transcription levels. **a** AGS cells treated with 75 and 150 μM Genipin for 24 h were harvested for western blotting with the indicated antibodies. **b** MKN45 (left) and SNU638 (right) cells were treated with 75 and 150 μM Genipin for 24 h. Mcl-1 protein level was detected by immunoblotting. **c**, **d** Total mRNA was isolated from AGS cells treated with 75 and 150 μM Genipin for 24 h. mRNA levels of Mcl-1 were measured by qRT-PCR (**c**) and RT-PCR (**d**). ** represents a statistically significant difference of *P* < 0.01. **e**–**g** AGS cells were transfected with Mcl-1 siRNA (siMcl-1). Genipin (150 μM) was treated with transfected cells for 24 h. Cell lysates were assessed by qRT-PCR (**e**), western blotting with cleaved PARP, Mcl-1, and β-actin (**f**) and flow cytometry (**g**). **h**–**j** AGS cells were transfected with His-Mcl-1 overexpression plasmid. Genipin (150 μM) was treated with transfected cells for 24 h. Protein levels of cleaved PARP, Mcl-1, and β-actin as well as Mcl-1 mRNA levels were assessed by qRT-PCR (**h**), immunoblotting (**i**) and flow cytometry (**j**). ***, *P* < 0.001

We detected the expression levels of pro-and anti-apoptotic proteins by western blot analysis in AGS cells to investigate whether Genipin regulates apoptotic proteins. We found that the Mcl-1 protein level was significantly decreased by Genipin treatment (Fig. 2a). Genipin also downregulated the protein levels of Mcl-1 in the other gastric cancer cell lines MKN45 and SNU638 (Fig. 2b). To determine whether Genipin affects the mRNA level of Mcl-1, RT-PCR and qRT-PCR assays were performed. As shown in Fig. 2c and d, the mRNA levels of Mcl-1 were decreased under Genipin-treated conditions, suggesting that Genipin regulates the transcription level of Mcl-1.

To confirm the association between Genipin-reduced Mcl-1 and apoptosis, we first overexpressed or knocked down Mcl-1 and administered Genipin treatment (Fig. 2e and h). Knockdown of Mcl-1 further increased apoptosis induced by Genipin, whereas apoptosis by Genipin was reduced by Mcl-1 overexpression (Fig. 2f, g, i, and j). Together, these results demonstrate that downregulation of Mcl-1 in Genipin-treated gastric cancer cells causes apoptosis.

### Genipin downregulates phosphorylated Stat3

**Fig. 3:**
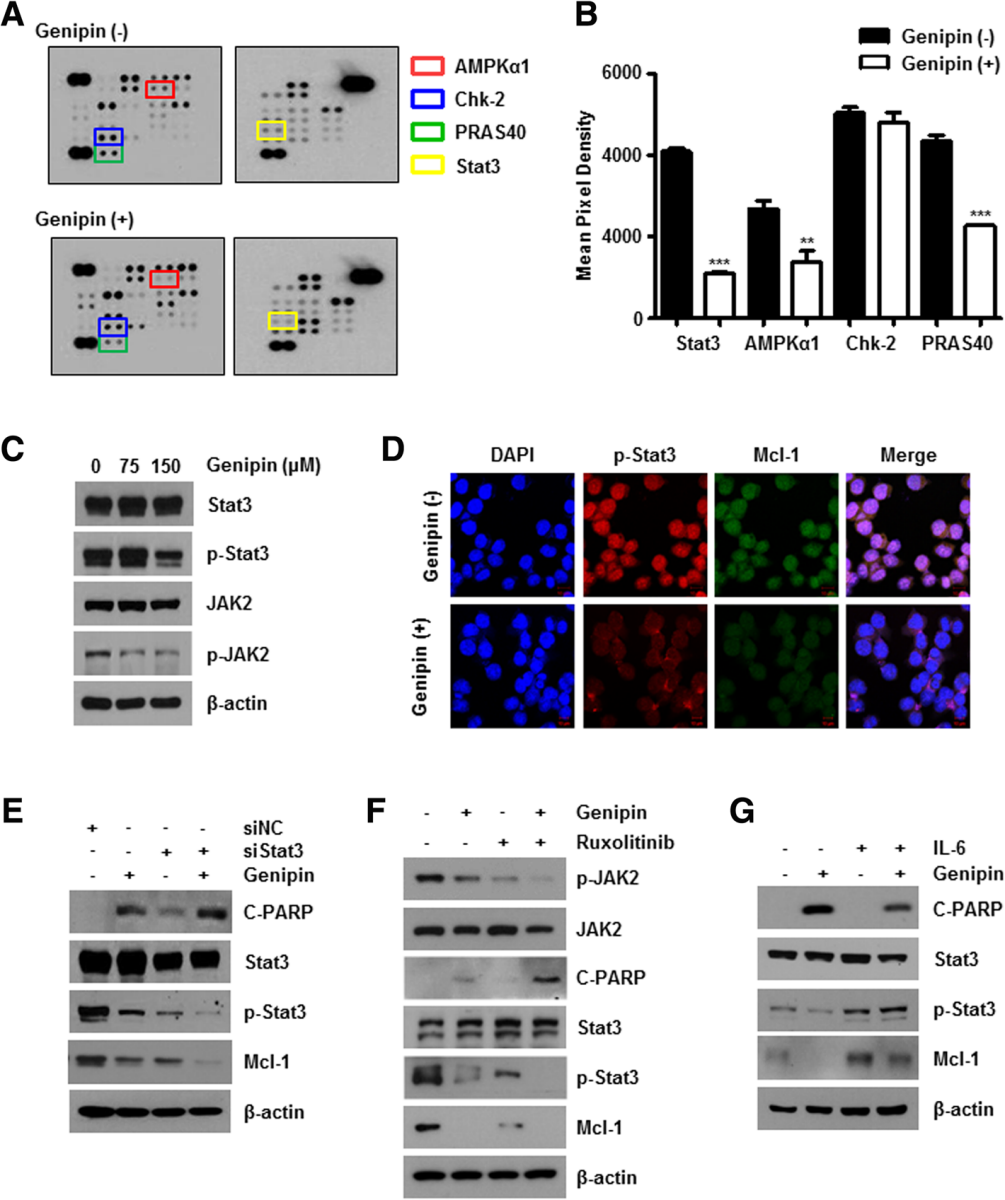
Treatment of Genipin affects apoptosis by downregulating phosphorylated Stat3. **a**, **b** AGS cells were treated with 150 μM Genipin for 24 h. Cell lysates were hybridized using the Proteome Profiler Phospho-Kinase Array kit (**a**). The graph represents quantification of mean pixel density using Image J (1.5 version) program (**b**). **c** Protein levels of Stat3, phospho-Stat3, JAK2, and phospho-JAK2 were confirmed by immunoblotting in AGS cells treated with Genipin for 24 h. β-Actin was used as a loading control. **d** AGS cells treated with or without 150 μM Genipin were immunostained with anti-p-Stat3 (red) and Mcl-1 (green). Images were captured using a confocal microscope. **e** AGS cells were transfected with Stat3 siRNA (siStat3). After incubation, transfected cells were treated with 150 μM Genipin for 24 h. Cell lysates were detected by western blotting with the indicated antibodies. **f** Cells were treated with 150 μM Genipin for 24 h following pre-treatment of 10 μM ruxolitinib for 1 h. Cell lysates were evaluated by western blotting with the indicated antibodies. **g** AGS cells were treated with 150 μM Genipin for 24 h following pre-treatment with 50 nM IL-6 for 30 min. Protein levels of Stat3, phosphorylated Stat3, Mcl-1, and cleaved PARP were assessed by immunoblotting

To evaluate specific effector signaling proteins affected by Genipin treatment, we examined phosphorylation using a protein kinase array. Genipin decreased the phosphorylation of several proteins, including AMPKα1 and particularly Stat3 (Fig. 3a and b). To confirm this result, western blot analysis was conducted. As shown in Fig. 3c, the protein levels of phosphorylated JAK2 and phosphorylated Stat3 were decreased after Genipin treatment. Additionally, Genipin decreased the fluorescence intensity of phospho-Stat3 and Mcl-1 (Fig. 3d). Since JAK-Stat3 signaling is known to regulate invasion and metastasis [24], we were examined whether Genipin affects invasion. As shown in Additional file 2: Figure S2, there was no significant difference in the expression of epithelial-mesenchymal transition (EMT)-related protein as well as the invasion ability of control and Genipin treated groups.

To determine the relationship between Stat3 reduction by Genipin and Mcl-1, AGS cells were transfected with Stat3 siRNA (siStat3) in the presence and absence of Genipin, and the level of Mcl-1 was analyzed by western blotting (Fig. 3e). Genipin significantly decreased Mcl-1 protein levels, and Stat3 knockdown further reduced the level of Mcl-1. Consistent with this, ruxolitinib, a JAK/Stat signaling pathway inhibitor, also decreased phospho-JAK2 and phospho-Stat3 protein levels (Fig. 3f). However, activation of the JAK/Stat pathway by IL-6 partially reversed Genipin-induced Mcl-1 attenuation (Fig. 3g). Moreover, the link between Stat3 inhibition and apoptosis in Genipin-treated AGS cells was examined by immunoblotting. We found that the Genipin-induced increase in cleaved PARP was partially reversed by Stat3 knockdown, whereas IL-6 decreased cleaved PARP, which was induced by Genipin (Fig. 3e–g), indicating that Genipin causes apoptosis through the JAK2/Stat3 signaling pathway.

### Genipin leads to mitochondrial dysfunction

**Fig. 4:**
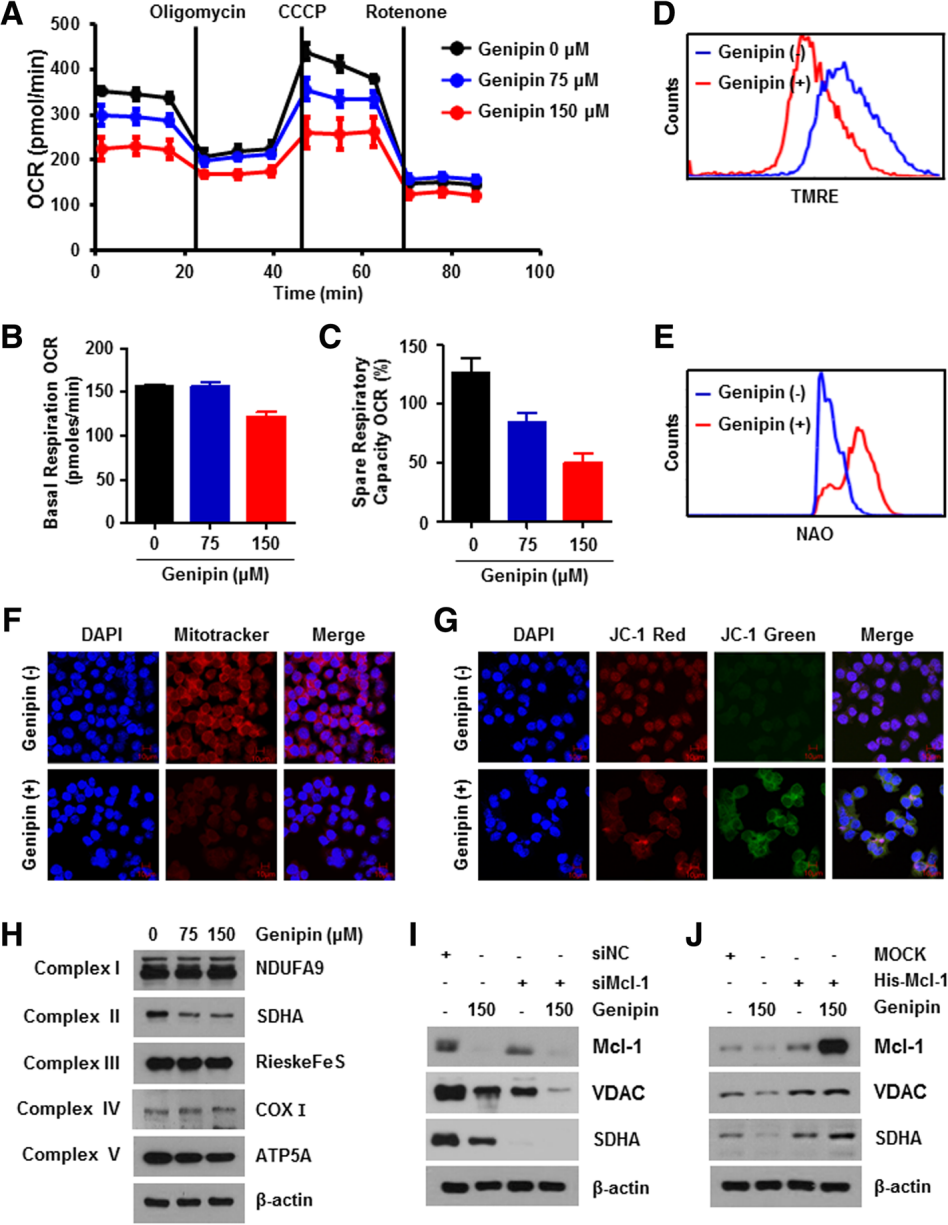
Genipin enhances mitochondria dysfunction through Mcl-1. **a**–**c** OCR was measured in AGS cells treated with Genipin using an XF analyzer. OCR levels were detected after addition of 2 μg/mL oligomycin, 5 μM m-chlorophenyl hydrazome, and 2 μM rotenone (**a**). The basal respiration OCR (**b**) and spare respiratory capacity (**c**) were quantified by OCR level. **d**, **e** AGS cells were treated with 150 μM Genipin for 24 h. Treated cells were stained with 10 μM TMRE (**d**) and 10 μM NAO (**e**) for 10 min at 37 °C. Stained cells were harvested and measured by flow cytometry. Fluorescence staining intensity was measured using flow cytometry. **f**, **g** 150 μM Genipin treated or untreated cells for 24 h were immunostained with Mitotracker (red) (**f**) and JC-1 (**g**). Images were captured using a confocal microscope. **h** Western blot analysis of mitochondrial electron transport chain proteins after Genipin treatment in AGS cells. **i** AGS cells were transfected with siMcl-1. Transfected cells were treated Genipin 150 μM for 24 h. MCl-1, VDAC, and SDHA protein levels were detected by western blotting with β-actin used as a loading control. **j** AGS cells were transfected with His-Mcl-1 plasmid. Mcl-1 overexpression cells were treated with 150 μM Genipin. Whole cell lysates were collected and incubated with the indicated antibodies

Because Mcl-1 is known to be highly associated with mitochondrial function [4, 5], we first examined the effects of Genipin on the function of the mitochondria using an XF24 analyzer. Genipin abolished the OCR as well as both basal respiration and spare respiratory capacity (Fig. 4a–c), while ECAR did not change (Additional file 3: Figure S3A and B). To further investigate mitochondrial dysfunction, we confirmed the number of mitochondria and MMP by staining the mitochondria with NAO and Mitotracker. As shown in Fig. 4e and f, the Mitotracker and NAO intensities were dramatically attenuated in response to Genipin exposure. Furthermore, we tested the decrease of MMP in Genipin-treated AGS cells using TMRE and JC-1 probe. JC-1 is a cationic carbocyanine dye that accumulates in the mitochondria. JC-1 monomers emit green fluorescence, while JC-1 aggregates emit red fluorescence [25]. Genipin treatment decreased the staining intensity of TMRE, but the JC-1 green fluorescence signal was increased by Genipin treatment (Fig. 4d and g). To assess the effect of Genipin on the mitochondrial electron transport chain complex, we confirmed the change in each mitochondrial electron transport chain complex protein by western blotting. The expression of SDHA, which indicates complex II, was decreased by Genipin treatment (Fig. 4h). To investigate the effect of Genipin-induced mitochondrial dysfunction on ROS production, we examined the generation of ROS in the mitochondria, the main site of ROS production. As shown in Additional file 3: Figure S3C, Genipin remarkably increased mitochondrial ROS generation. Furthermore, we investigated relationship between Mcl-1 downregulation induced by Genipin and mitochondrial function, we transfected with siRNA for Mcl-1 or an Mcl-1 overexpression vector (His-Mcl-1), Knockdown of Mcl-1 further reduced VDAC, which reveals the number of mitochondria, and SDHA be Genipin, whereas Genipin-induced a decrease in SDHA and VDAC when Mcl-1 was overexpressed (Fig. 4i and j). Collectively, these findings demonstrate that the decrease of Mcl-1 by Genipin caused mitochondrial dysfunction by decreasing the mitochondria number and mitochondrial complex II activity.

## Discussion

The chemotherapeutic agents currently used for cancer treatment are limited because of their various side effects [26]. Therefore, cancer prevention and treatment with natural products that are safe and have low toxicity is a powerful therapeutic strategy for cancer [14, 27]. Our studies focused on the apoptotic effects of Genipin, a natural-derived compound. At present, natural-derived compounds including plants induce cytotoxicity of cancer cells, but none of them are under clinical trials because of various side effects. It is particularly a problem that it is not known how certain components of the compound work and cause side effects. So the purpose of our study is to identify the cytotoxicity ability of a single component and to help develop new drugs. In additionally, Genipin works well on acidic condition. Chitosan microspheres used for the treatment of Helicobacter pylori gastric infection capture and remove bacteria by crosslinking with bacteria (muco/bacterial adhesion). At acidic pH, this crosslinking became unstable, but Genipin enhanced crosslinking, which did not dissolve the chitosan microspheres and increased residence time at the stomach [28, 29]. The influence of Genipin on cancer cell apoptosis has been widely reported previously. However, the exact mechanism of apoptosis induced by Genipin remained unclear, and few studies have evaluated Genipin-induced apoptotic cell death, particularly in gastric cancer. In this study, we showed for the first time that Genipin leads to apoptosis by downregulating Mcl-1 through JAK2/Stat3.

We found that Genipin caused apoptotic cell death via both intrinsic (caspase 3 and PARP) and extrinsic (caspase 8) pathways. Moreover, Genipin elevated the Sub-G1 proportion. Genipin also reduced the protein levels of the anti-apoptotic Bcl-2 family member Mcl-1. Mcl-1 is crucial for homeostasis, has a short half-life, and is tightly regulated at the mRNA and protein levels [30, 31]. In many cancers including gastric cancer, Mcl-1 is overexpressed and is associated with patient survival and tumor progression [32–35]. Our data showed that Mcl-1 overexpression inhibited Genipin-induced apoptosis, whereas Mcl-1 knockdown induced apoptosis by Genipin, suggesting that downregulation of Mcl-1 is essential for Genipin-induced apoptosis.

According to the Warburg effect, it affects cell growth of cancer cells is by regulating ATP production through the mitochondrial respiratory chain by aerobic glycolysis [36]. Interestingly, Genipin did not affect the glycolysis of gastric cancer cells. However, we showed that Genipin decreased OCR and respiration in a dose-dependent manner. Moreover, proton leakage and ATP production were also inhibited by Genipin treatment (data not shown). These findings indicate that, Genipin reduces energy production by reducing oxygen consumption without affecting glycolysis through lactate generation. Although Genipin is known to affect mitochondrial function through uncoupling protein 2 [37, 38], little is known about its effect on the mitochondrial complex. Genipin has been reported to reduce the activity of mitochondrial complexes I and III; however, in our system, Genipin also decreased the expression levels of the mitochondrial complex II protein SDHA and mitochondrial complex V protein ATP synthase subunit alpha, as well as electron turnover (data not shown), indirectly suggesting that Genipin modulates mitochondrial complexes II and V. In addition, Genipin diminished MMP. During apoptosis, a reduction in MMP causes conformational changes of the mitochondria through matrix condensation (eg, cardiolipin) and enables cytochrome *c* to be released more easily from the cristae into the intermembrane space [39]. The MMP can be reduced by cytosol acidification. Therefore, Genipin may lead to a decrease in MMP by increasing cytosol acidification [40]. In additionally, mitochondrial ROS production is increased by Genipin. Mitochondrial dysfunction associated with ROS production. For instance, NADH accumulation and induction of RAS recruitment to mitochondria can reduce ROS by reducing antioxidant enzymes, indicating that Genipin may elevate ROS generation by abolishing antioxidant enzymes [41]. Genipin-reduced cardiolipin and SDHA are present in the mitochondrial inner membrane, suggesting that Genipin induces internally mitochondrial dysfunction rather than externally. Mcl-1 has a different isoform depending on its location in the mitochondria, and plays a role as an anti-apoptotic molecule in the outer mitochondrial membrane. However, in the inner mitochondrial membrane, the Mcl-1 isoform is a truncated form of the amino terminus and is important for mitochondrial functions such as cristae ultrastructure, mitochondria fusion, respiration, ATP production, membrane potential, and maintenance of oligomeric ATP synthase [3]. Moreover, Mcl-1 overexpression further reduced the levels of SDHA decreased by Genipin, while restoring the SDHA protein levels affected by Mcl-1 knockdown. Although additional experiments are needed, we demonstrated that Genipin-induced Mcl-1 reduction causes mitochondrial dysfunction such as mitochondrial complex II / V activity, ATP production, and MMP inhibition.

Cytokine receptors without an intrinsic protein kinase domain transmit signals downstream, including Stats, through the JAK family (JAK1–3, and tyrosine kinase 2) [42]. The JAK family phosphorylates the tyrosine residue of the transcription factor Stat, which enable its binding to the promoter of target genes related to survival and apoptosis [43]. Intrinsic regulation such as post-translational modification and inhibition through the pseudokinase domain affects JAK activity. JAK activity is also regulated by extrinsic regulatory factors including phosphatases (Src homology 2 domain-containing phosphatase (SHPs), T-cell protein tyrosine phosphatase, CD45), SH2 domain-containing proteins (suppressors of cytokine signaling, SOCSs) and lymphocyte adapter protein [42]. Moreover, Genipin has been reported to regulate the JAK/Stat pathway by activating SHP1 and SOCS3 [44, 45], indicating that Genipin inhibits JAK activity by stimulating SHP1 and SOCS3.

## Conclusions

**Fig. 5:**
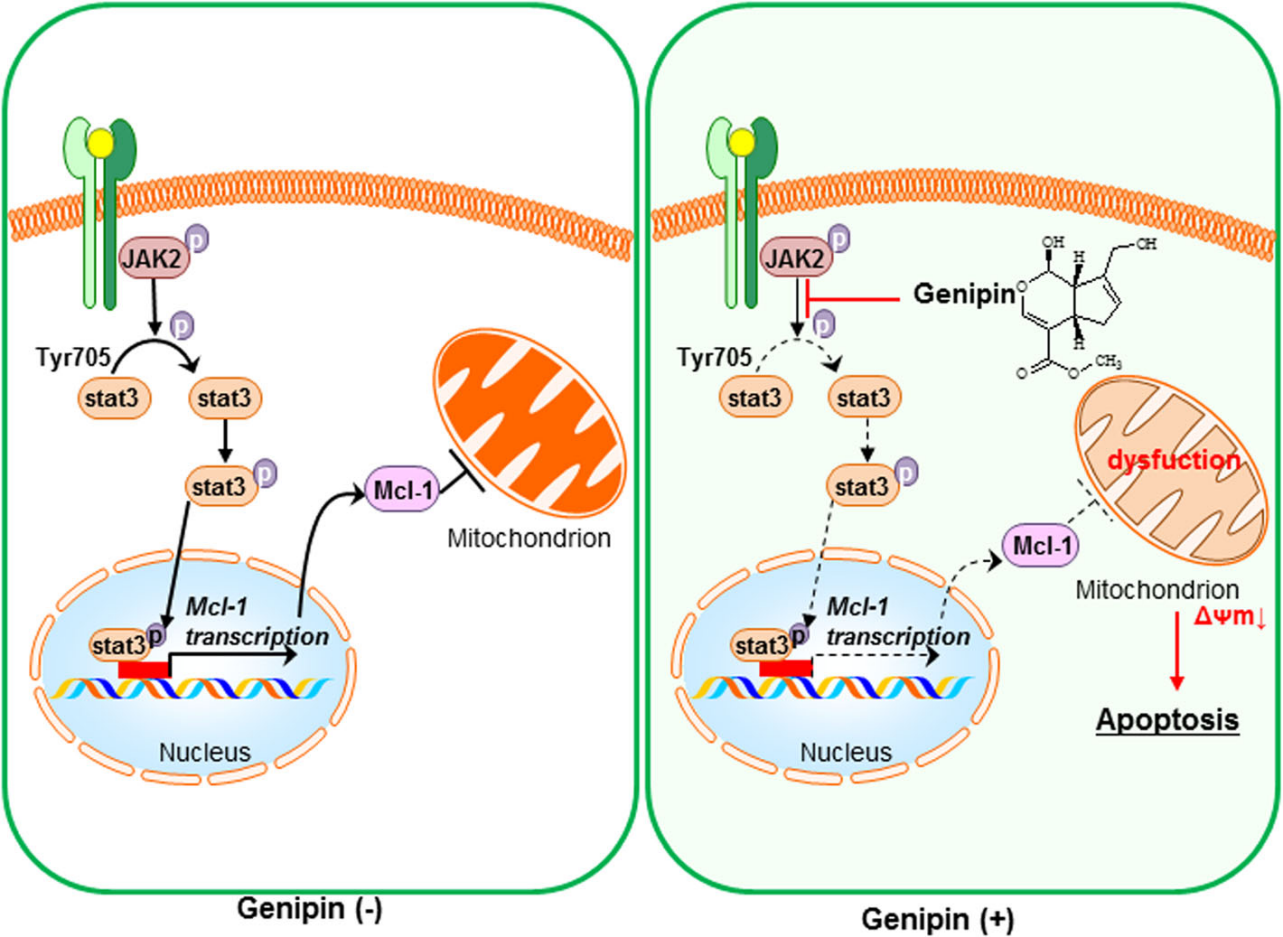
Schematic diagram of Genipin-mediated apoptosis mechanism

We found that Genipin induced apoptotic cell death in gastric cancer cell lines. This effect occurred because of mitochondrial dysfunction caused by decreased expression of Mcl-1 through the JAK/Stat3 pathway (Fig. 5). Thus, our study suggests that Genipin is useful as a new therapeutic agent for gastric cancer targeting JAK/Stat3 and Mcl-1.

## Additional files

Additional file 1: **Figure S1.** Genipin elevates the Sub-G1 population. (A) Genipin-treated AGS cells were stained with PI and analyzed by flow cytometry. (TIF 74 kb)

Additional file 2: **Figure S2.** Genipin does not affect invasion of gastric cancer cells. (A) AGS cells were seeded on Matrigel-coated upper chamber and incubated in serum free medium with or without Genipin for 48 h. Then, the number of cells captured with light microscopy (upper) and quantified graph (lower). (B) AGS cells treated with or without Genipin for 24 h were harvested for western blotting with the EMT-related antibodies. (TIF 192 kb)

Additional file 3: **Figure S3.** Genipin is not associated with glycolysis of gastric cancer cells. (A-B) Genipin was treated with AGS cells and analyzed by XF24 analyzer (A). The graph is the number of glycolysis quantified by ECAR (B). (C) 150 μM Genipin treated cells for 24 h were stained with MitoSOX. Then, the cells were analyzed by flow cytometry. (TIF 85 kb)

## Data Availability

The datasets used and/or analysed during the current study available from the corresponding author on reasonable request.

## Abbreviations

2P/S: penicillin and the streptomycin
Bcl-2: B-cell lymphoma 2
Bcl-xL: B-cell lymphoma-extra large
COXI: Cytochrome c oxidase I
DAPI: 4′,6-diamidino-2-phenylindole
DMEM: Dulbecco’s Modified Eagle’s Medium
FBS: Fetal bovine serum
FITC: Fluorescein isothiocyanate
HRP: Horseradish peroxidase
IL-6: Interleukin 6
JAK: Janus kinase
Mcl-1: Myeloid cell leukemia-1
MMP: Mitochondrial membrane potential
NAO: 10-N-nonyl acridine orange
NDUFA9: NADH dehydrogenase (ubiquinone) 1 alpha subcomplex subunit 9
OCR: Oxygen consumption rate
PBS: Phosphate buffered saline
PI: Propidium iodide;
qRT-PCR: quantitative real-time PCR
RieskeFeS: Rieske iron-sulfur
RT: Room temperature
RT-PCR: Reverse transcription-polymerase chain reaction
SDHA: Succinate dehydrogenase complex flavoprotein subunit A
SHP: Src homology 2 domain-containing phosphatase
siRNA: small interference RNA
SOCS: Suppressors of cytokine signaling
Stat3: Signal transducer and activator of transcription 3
TMRE: Tetramethylrhodamine ethyl ester
TUNEL assay: TdT-mediated dUTP nick-end labeling assay
VDAC: Voltage-dependent anion channel
