# Supplementary figures and images for: Genipin induces mitochondrial dysfunction and apoptosis via downregulation of Stat3/mcl-1 pathway in gastric cancer
Source: BMC Cancer. 2019 Jul 27;19:739. doi: 10.1186/s12885-019-5957-x (PMC6661087; doi:10.1186/s12885-019-5957-x)

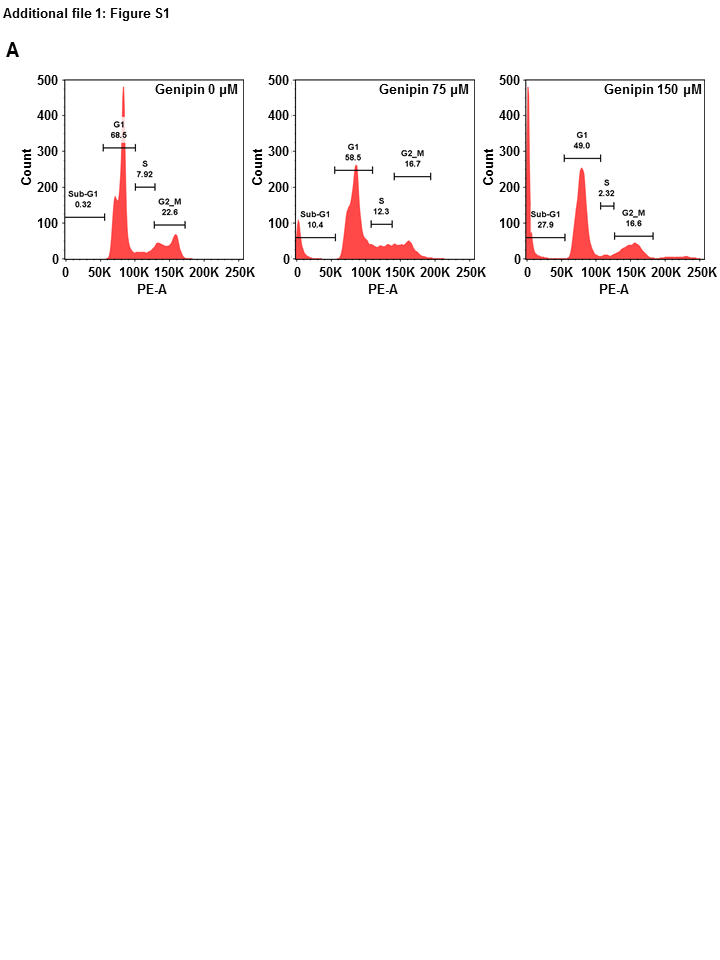

Supplement: Supplementary file 1 — Figure S1. Genipin elevates the Sub-G1 population. (A) Genipin-treated AGS cells were stained with PI and analyzed by flow cytometry. (TIF 74 kb) [file 12885_2019_5957_MOESM1_ESM.tif]

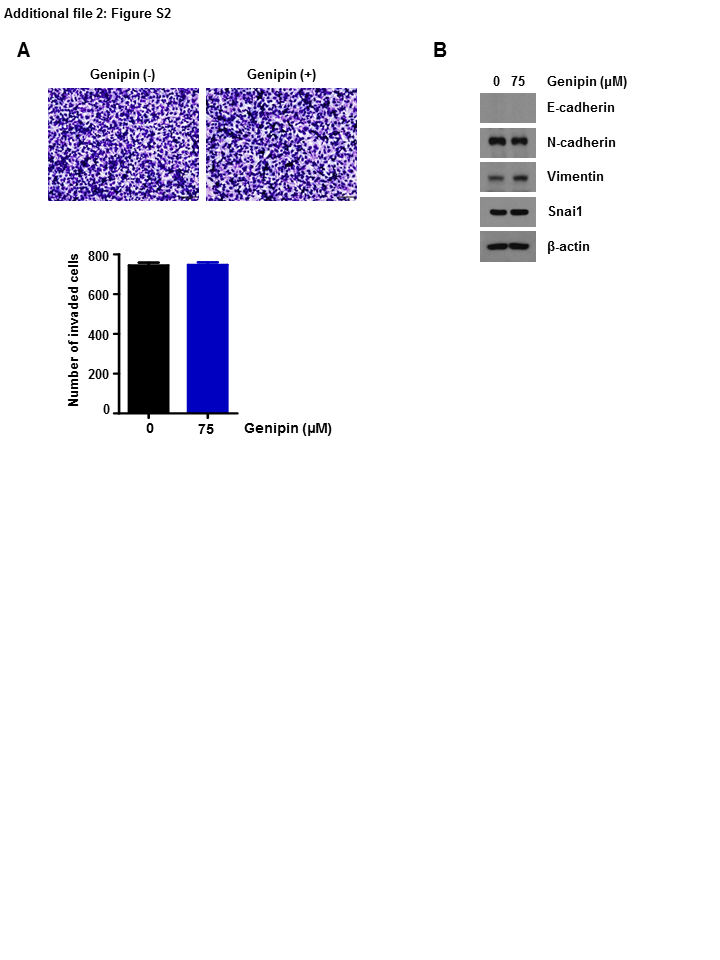

Supplement: Supplementary file 2 — Figure S2. Genipin does not affect invasion of gastric cancer cells. (A) AGS cells were seeded on Matrigel-coated upper chamber and incubated in serum free medium with or without Genipin for 48 h. Then, the number of cells captured with light microscopy (upper) and quantified graph (lower). (B) AGS cells treated with or without Genipin for 24 h were harvested for western blotting with the EMT-related antibodies. (TIF 192 kb) [file 12885_2019_5957_MOESM2_ESM.tif]

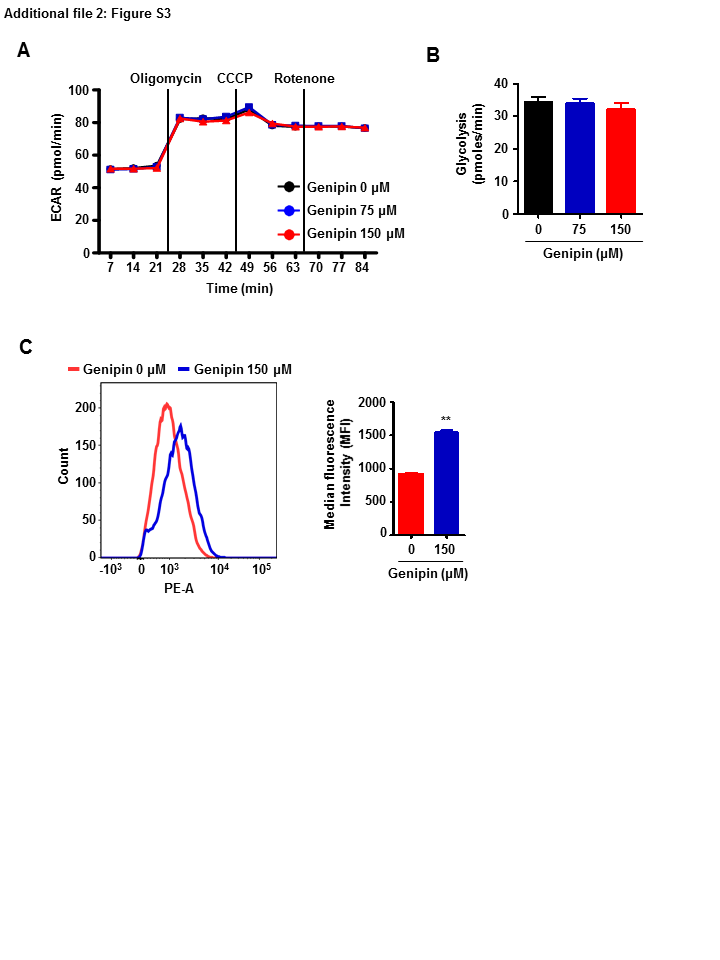

Supplement: Supplementary file 3 — Figure S3. Genipin is not associated with glycolysis of gastric cancer cells. (A-B) Genipin was treated with AGS cells and analyzed by XF24 analyzer (A). The graph is the number of glycolysis quantified by ECAR (B). (C) 150 μM Genipin treated cells for 24 h were stained with MitoSOX. Then, the cells were analyzed by flow cytometry. (TIF 85 kb) [file 12885_2019_5957_MOESM3_ESM.tif]
